# Supplementary figures and images for: A novel class of polymeric fluorescent dyes assembled using a DNA synthesizer
Source: PLoS One. 2020 Dec 4;15(12):e0243218. doi: 10.1371/journal.pone.0243218 (PMC7717558; doi:10.1371/journal.pone.0243218)

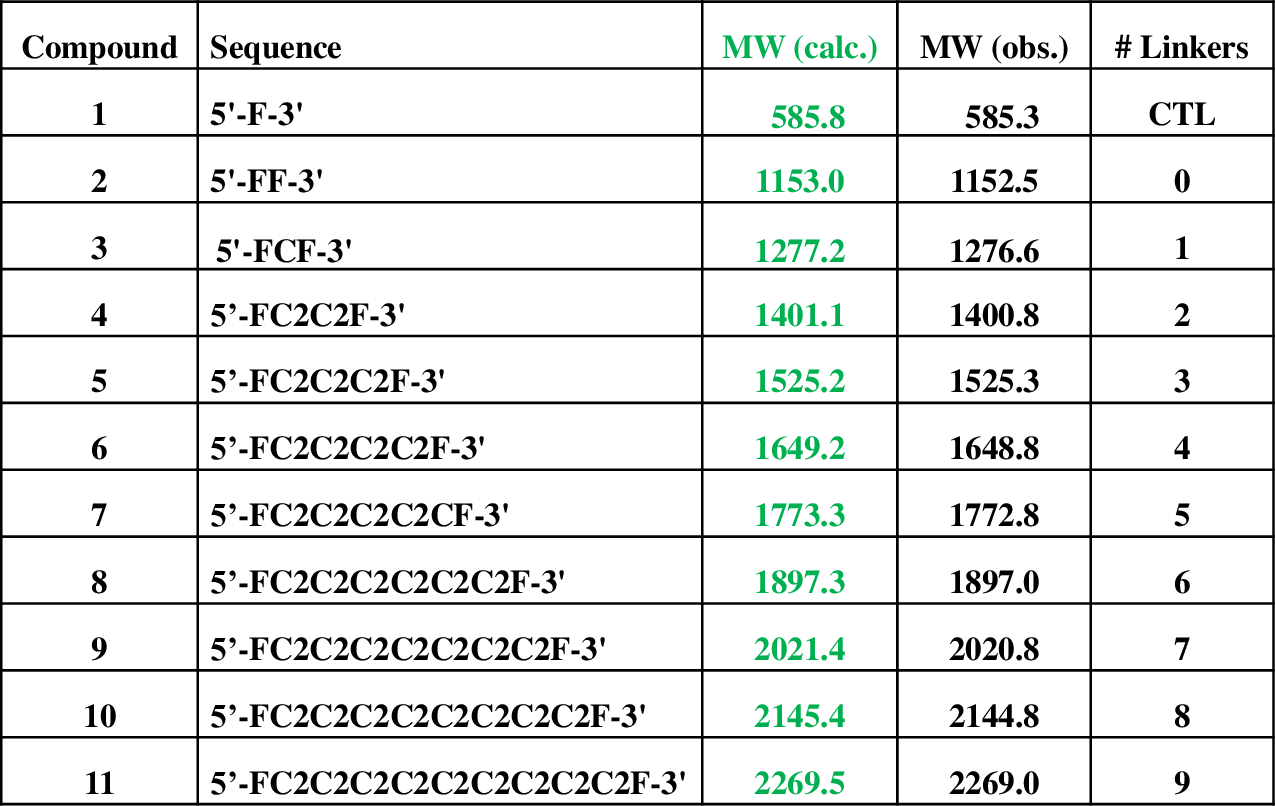

Supplement: S1 Fig — (TIF) [file pone.0243218.s001.tif]

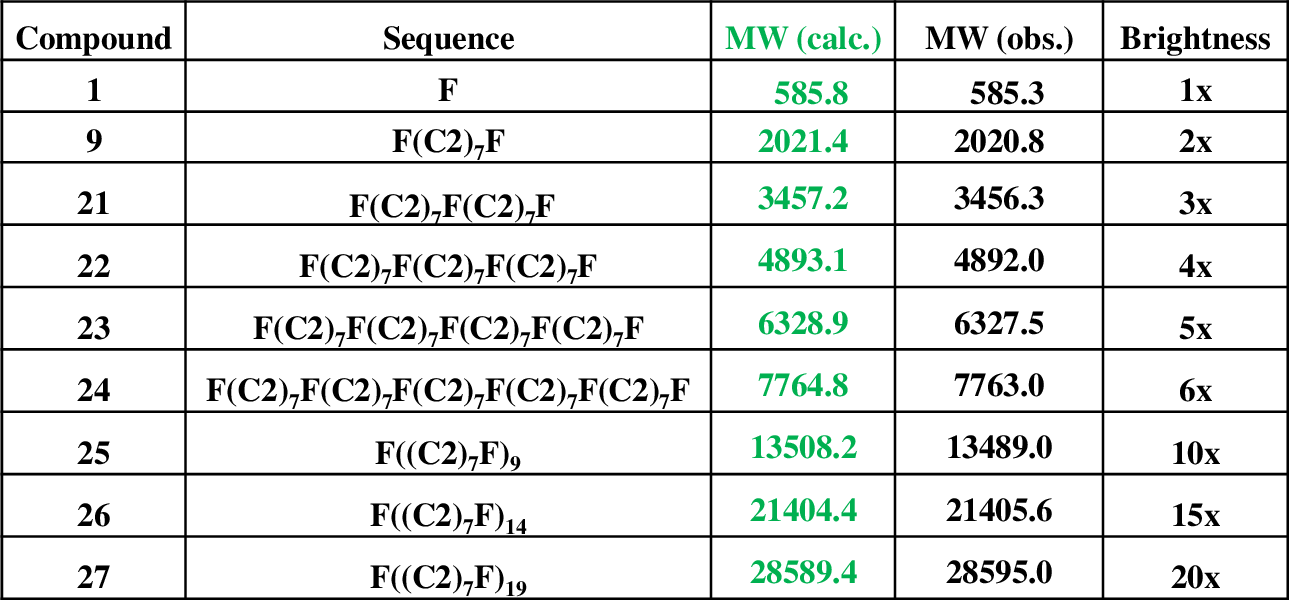

Supplement: S2 Fig — (TIF) [file pone.0243218.s002.tif]

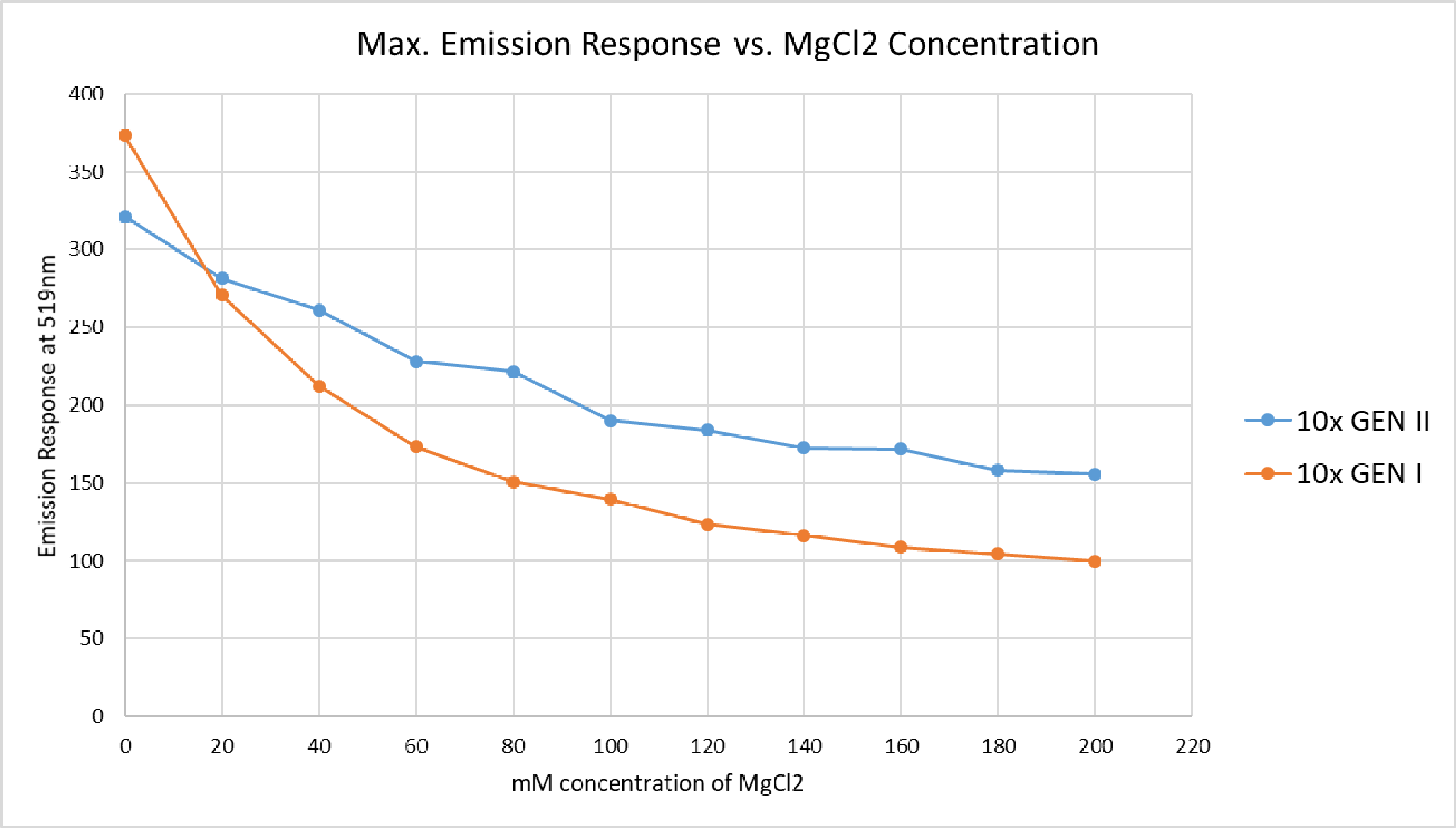

Supplement: S3 Fig — The presence of divalent MgCl2 had a significant downward effect on the emissions of both GEN I and GEN II polymer systems, but the degree and regularity of the effect was different between the two constructs: Where the emission values for GEN I (presumably the more rigid construct) fell more sharply, they also fell in a more uniform fashion, whereas the emission values for GEN II were less uniform and with a less drastic drop in efficiency. (TIF) [file pone.0243218.s003.tif]

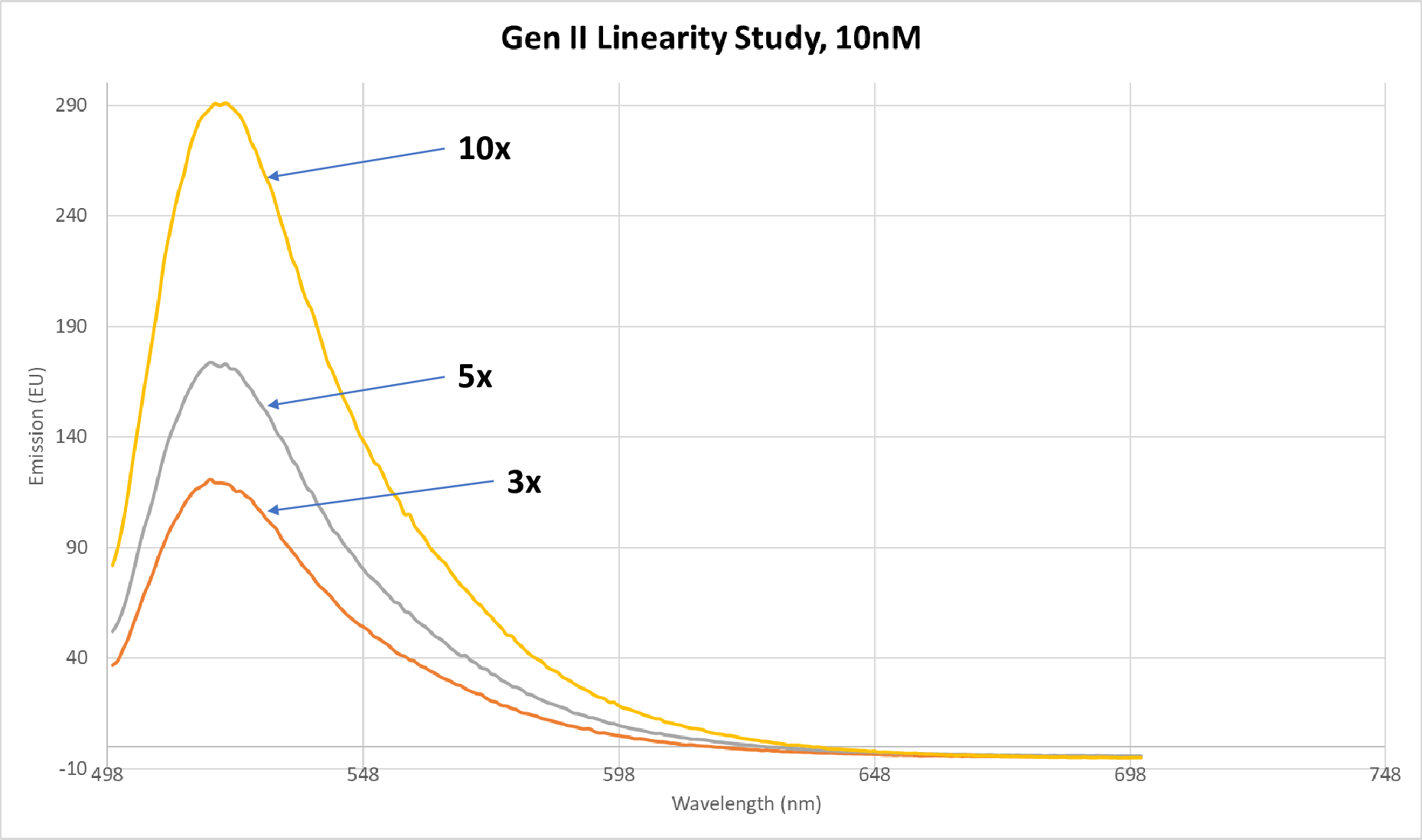

Supplement: S4 Fig — (TIF) [file pone.0243218.s004.tif]

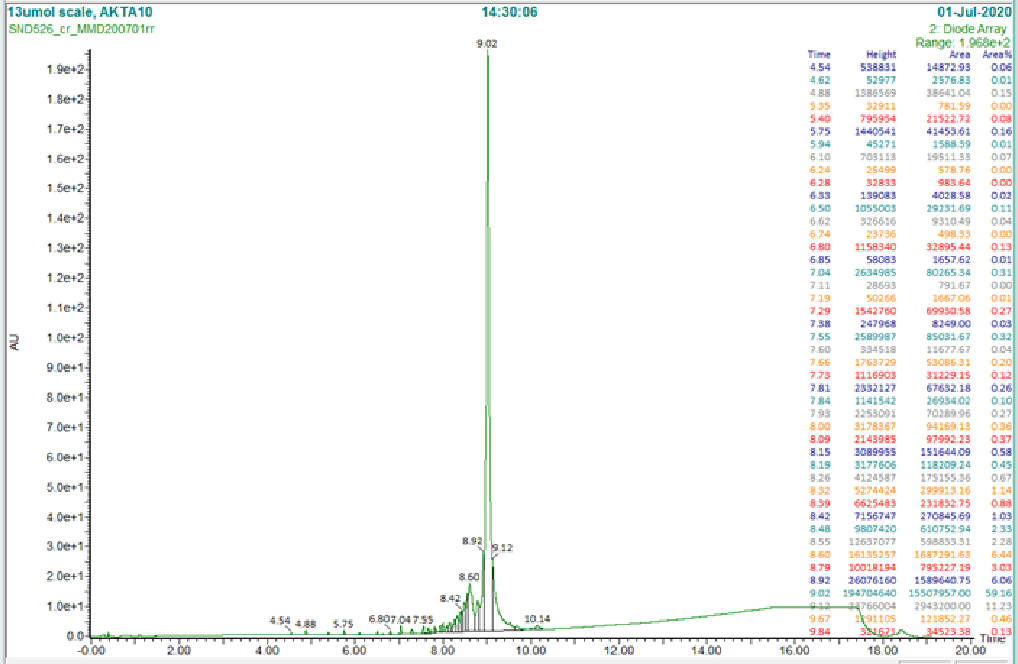

Supplement: S5 Fig — (TIF) [file pone.0243218.s005.tif]

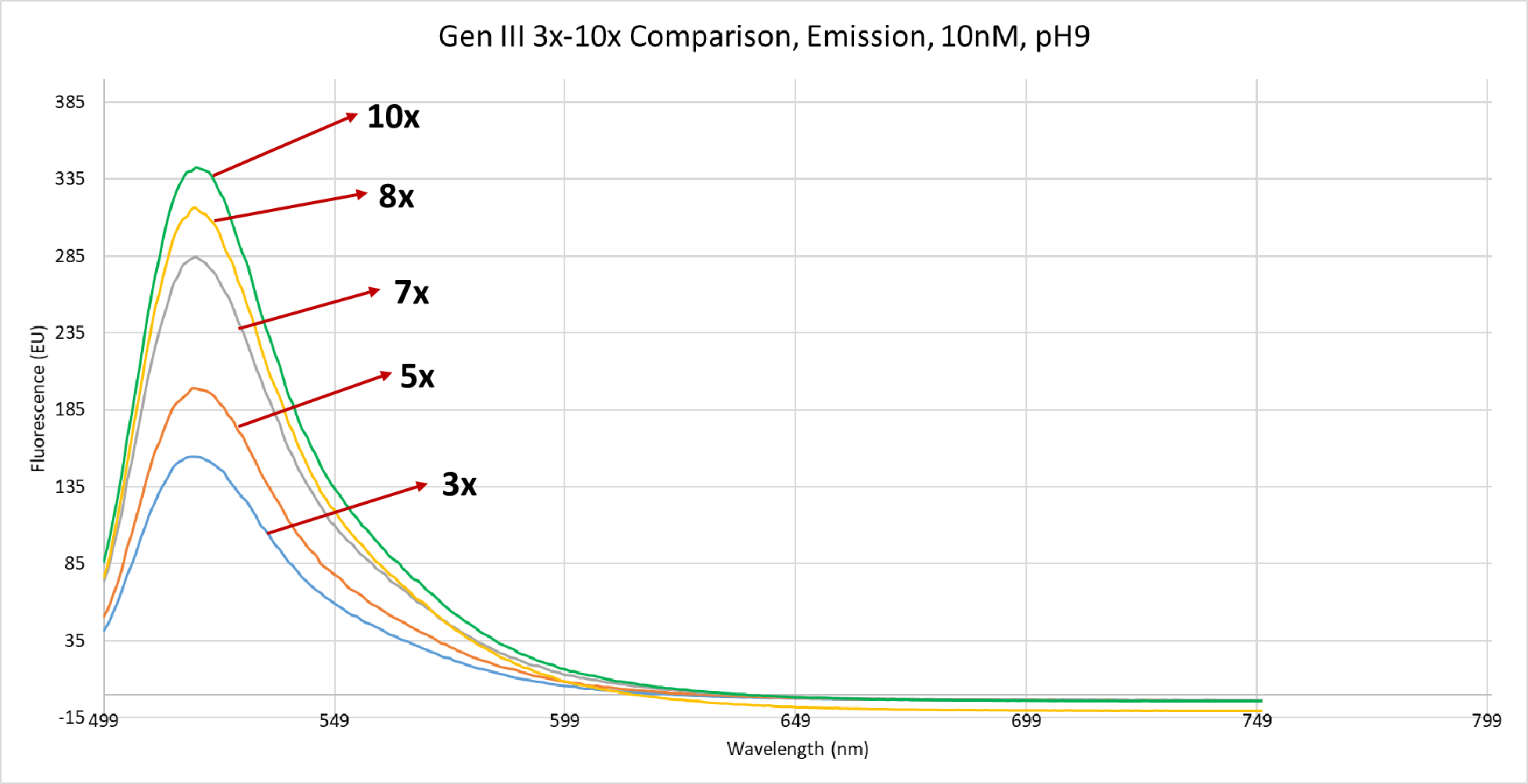

Supplement: S6 Fig — (TIF) [file pone.0243218.s006.tif]

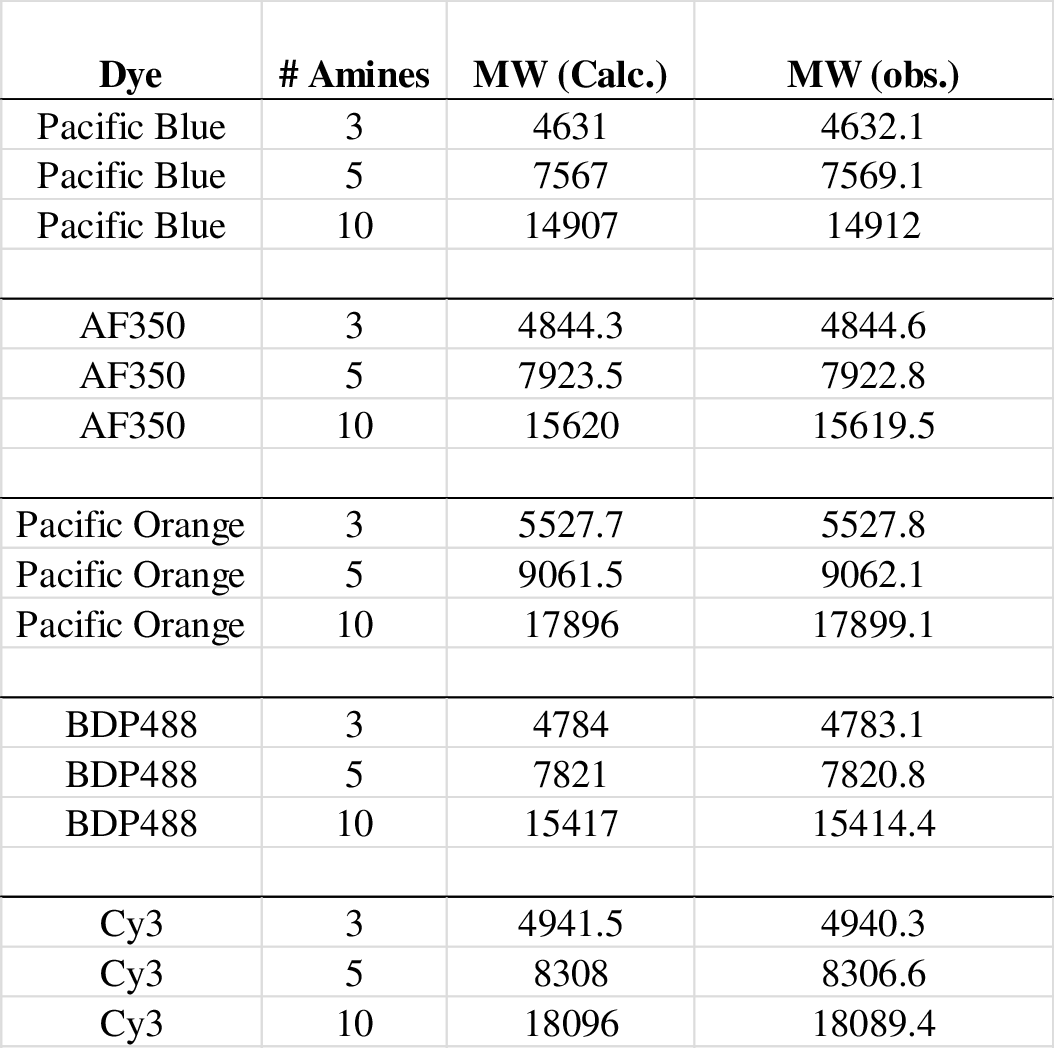

Supplement: S7 Fig — (TIF) [file pone.0243218.s007.tif]
