# Supplementary material for: A novel class of polymeric fluorescent dyes assembled using a DNA synthesizer
Source: PLoS One. 2020 Dec 4;15(12):e0243218. doi: 10.1371/journal.pone.0243218 (PMC7717558; doi:10.1371/journal.pone.0243218)
Supplement: S1 File — (DOCX) [file pone.0243218.s009.docx]

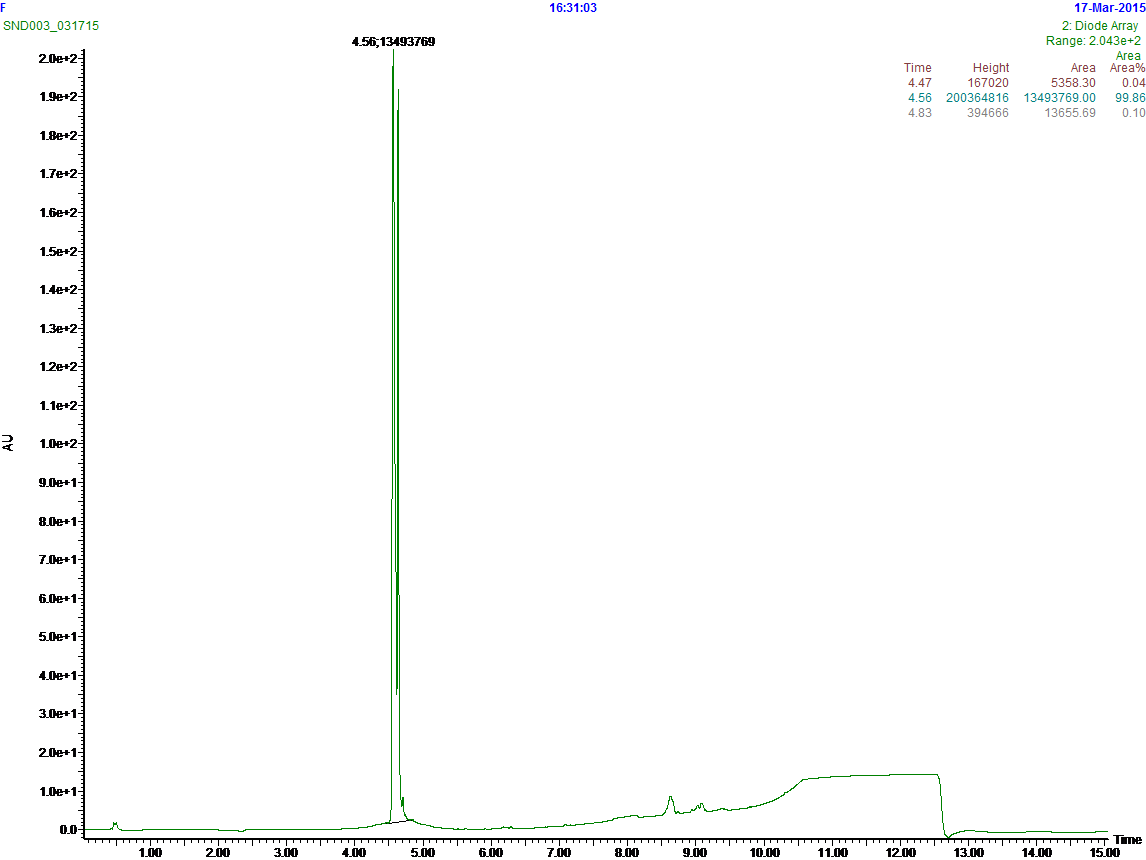


**Compound 1** - both peaks have the same MW, so overall purity is >99% by UPLC

**Compound 1** - Predicted MW is 585.8. Observed MW is 585.0.


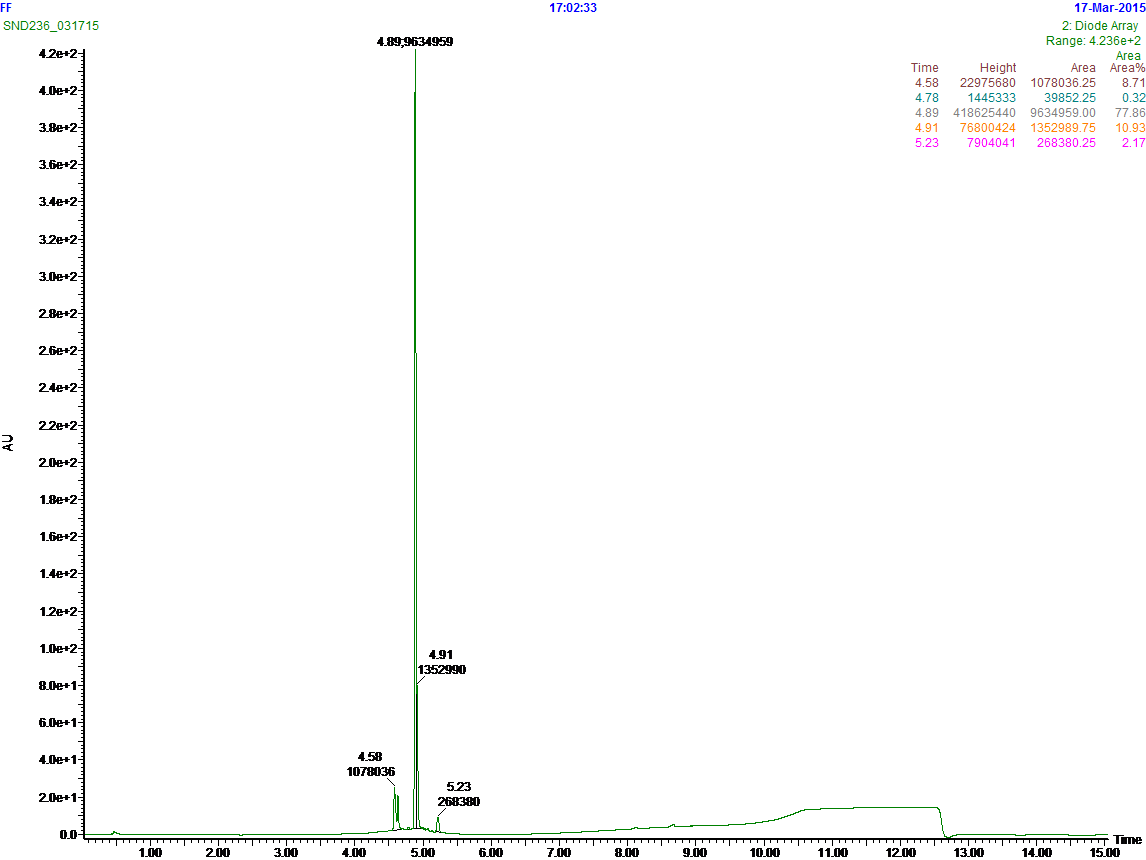


**Compound 2** - Overall purity by LC-DAD is 77.9%.

**Compound 2 -** Predicted MW is 1153.2. Observed MW is 1152.8.


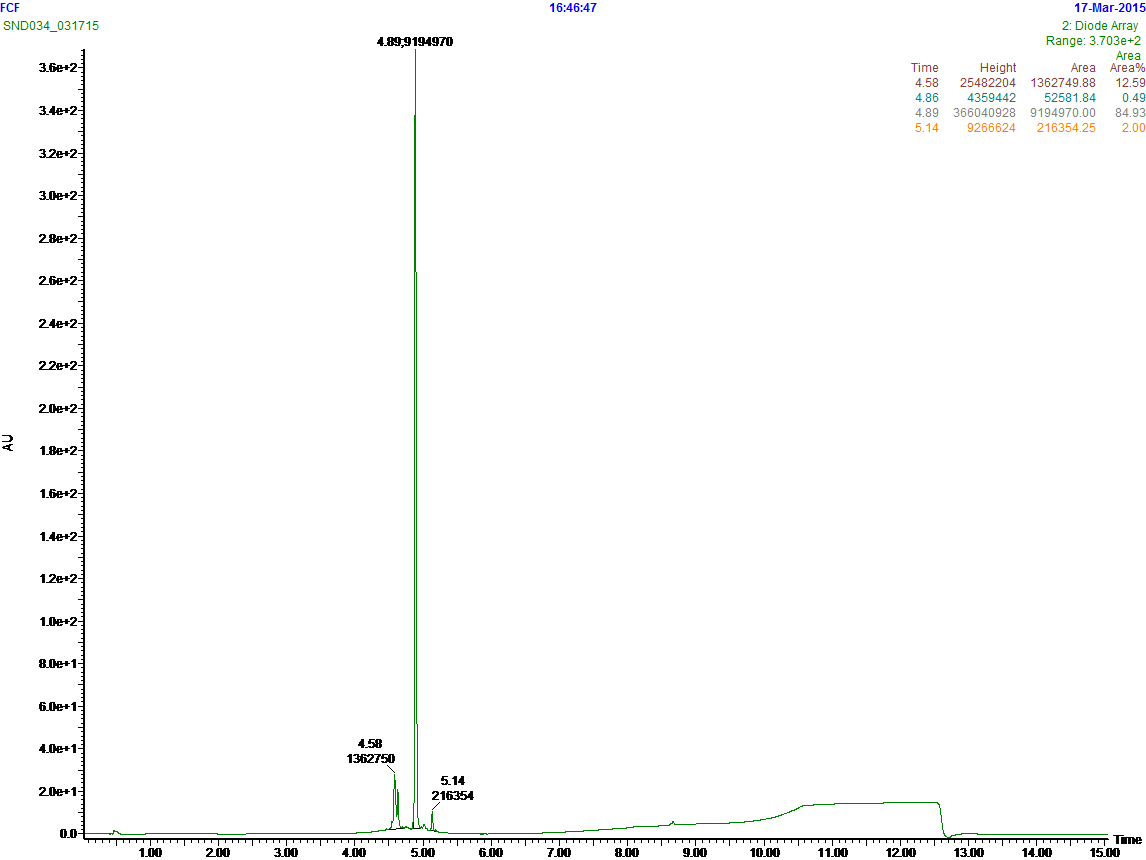


**Compound 3** - Overall purity is ~85% by LC-DAD.

 **Compound 3** - Predicted MW is 1277.2. Observed MW is 1276.7.


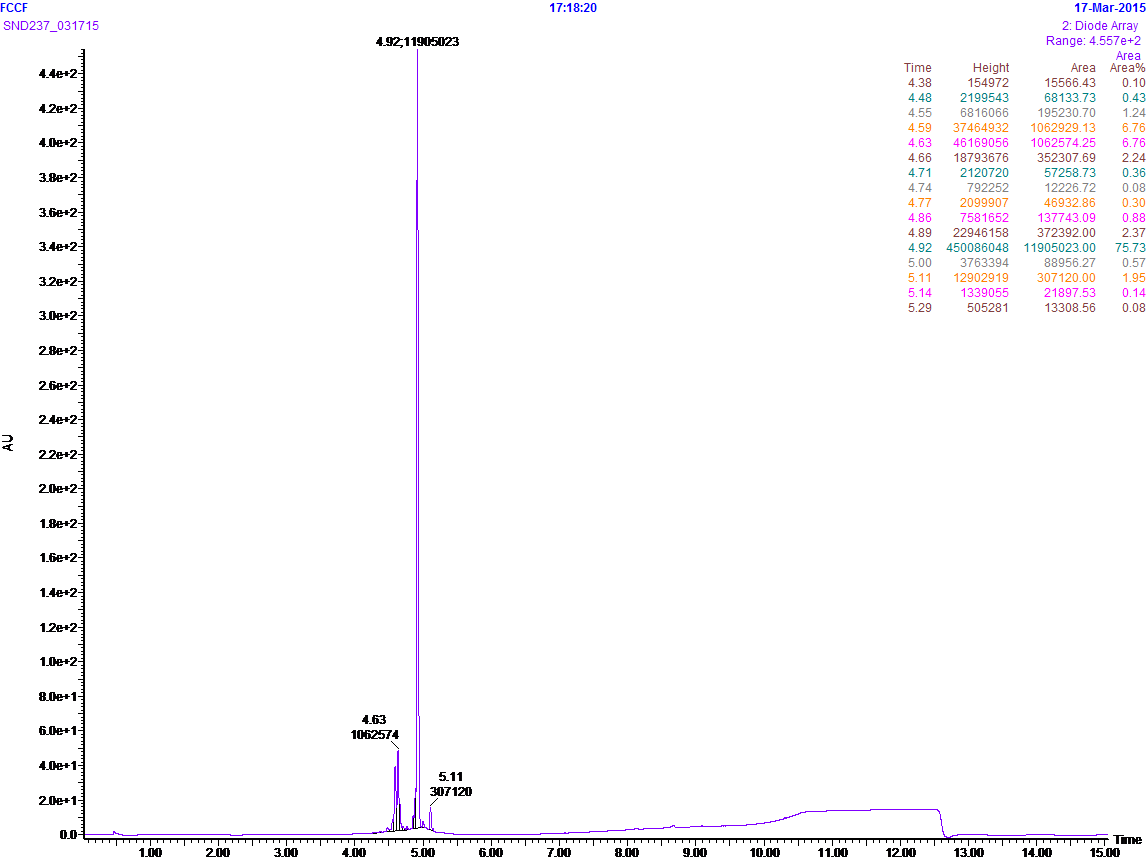


**Compound 4** - Overall purity is ~76%.

**Compound 4 -** Predicted MW is 1400.8. Observed MW is 1401.1.


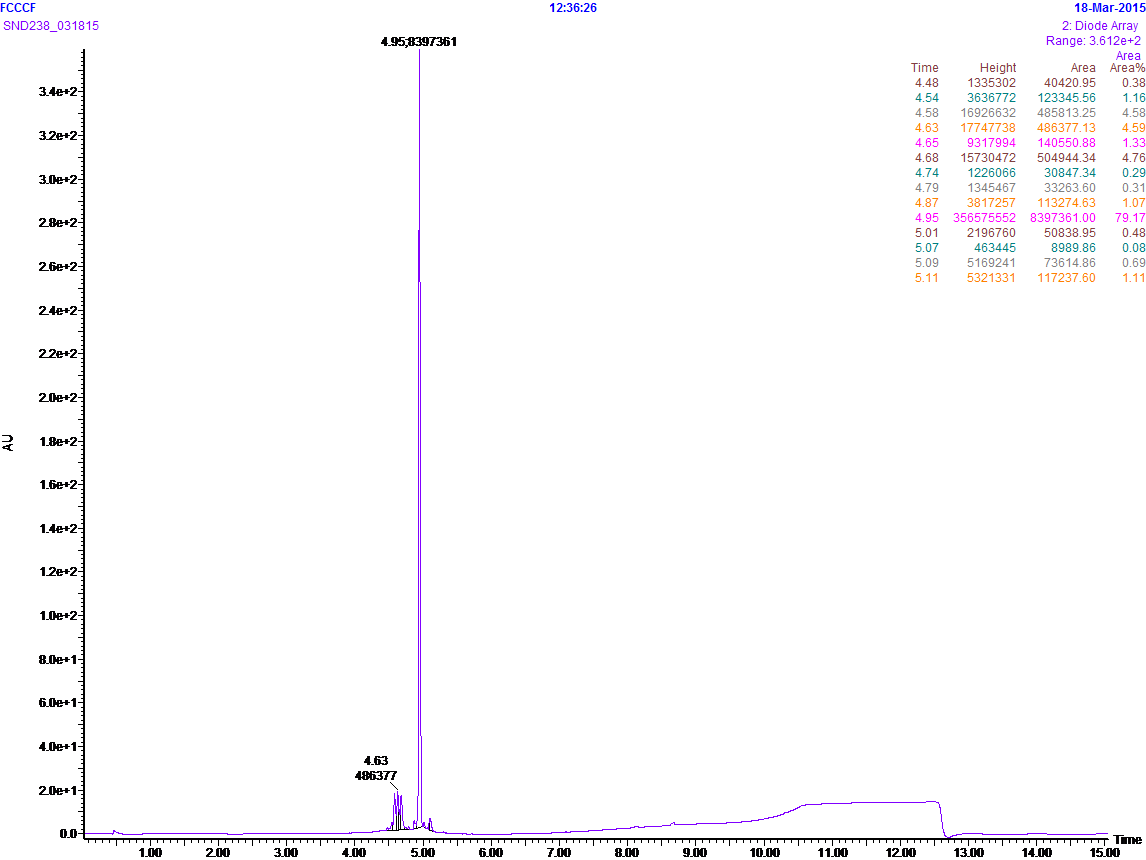


**Compound 5** - Overall purity by LC-DAD is 79%.

**Compound 5** - Predicted MW is 1524.4. Observed MW is 1525.0.


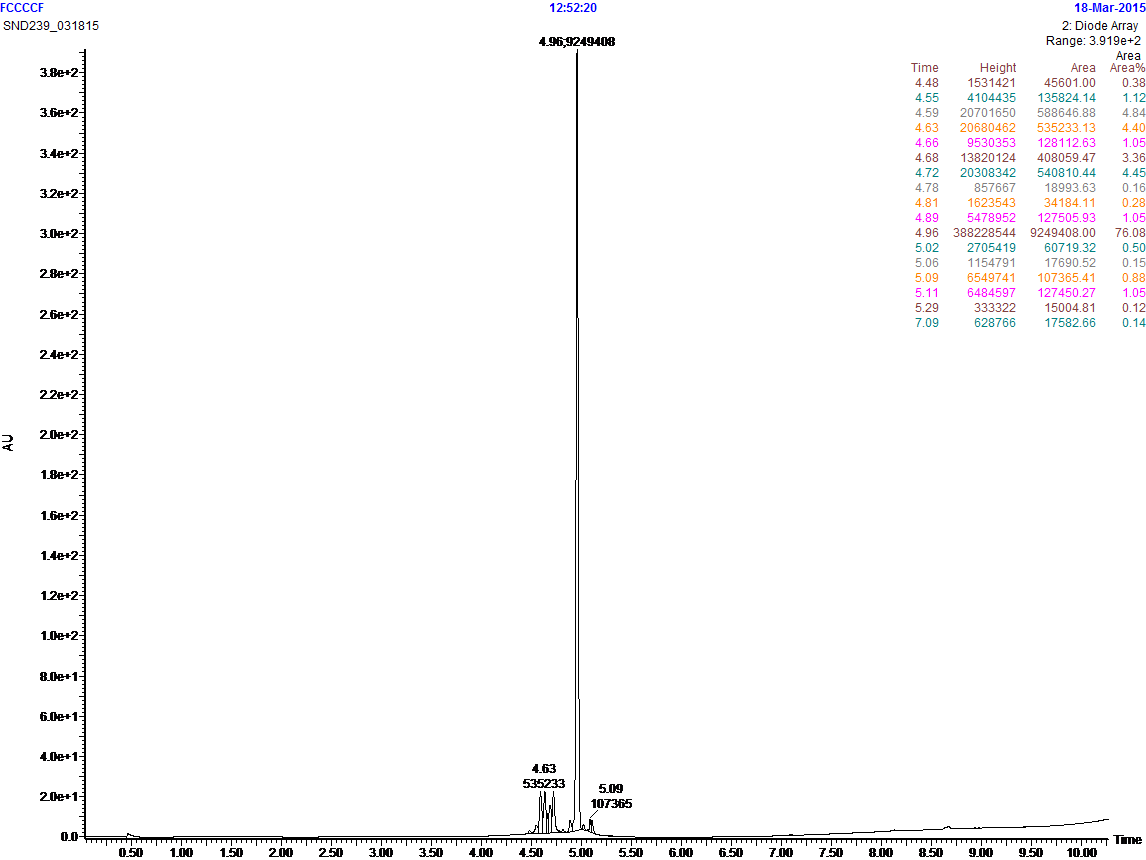


**Compound 6** - Overall purity by LC-DAD is 76%.

**Compound 6** - Predicted MW is 1648.0. Observed MW is 1648.9.


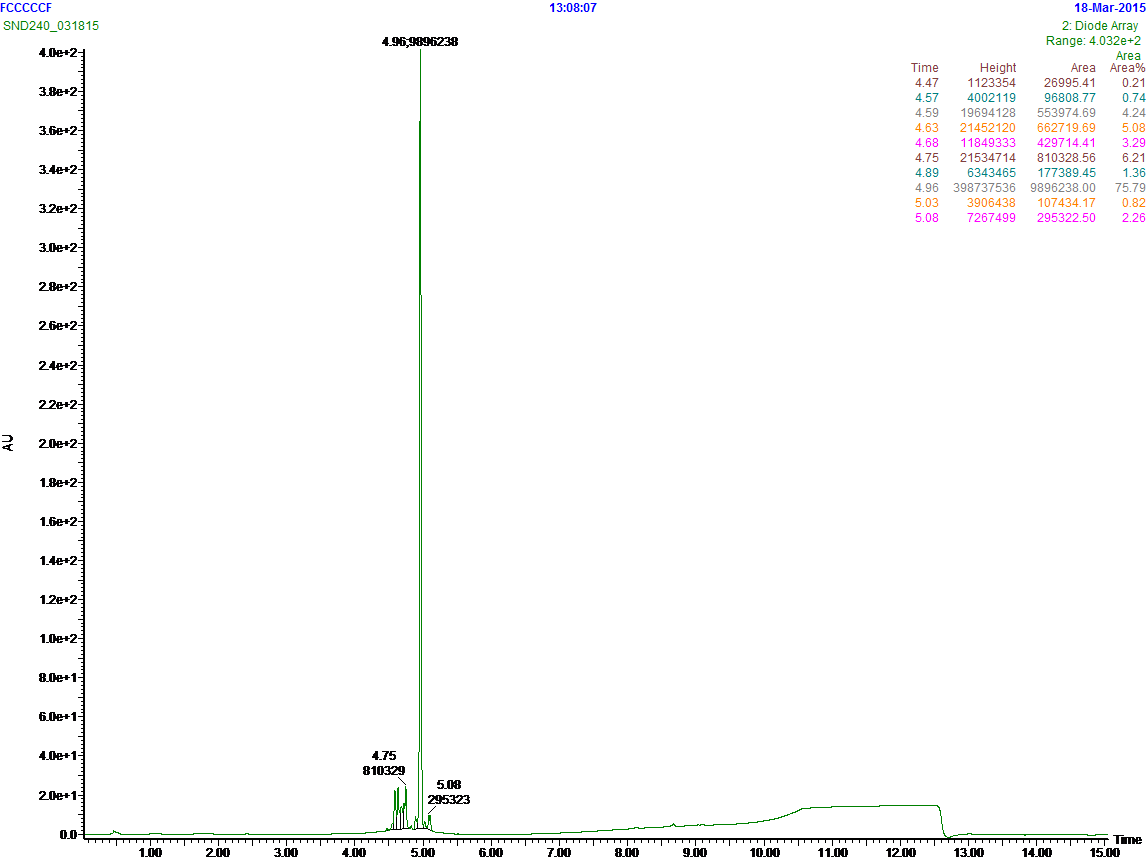


**Compound 7** - Overall purity by LC-DAD is ~76%.

**Compound 7** – Predicted MW is 1773.3. Observed MW is 1772.8.


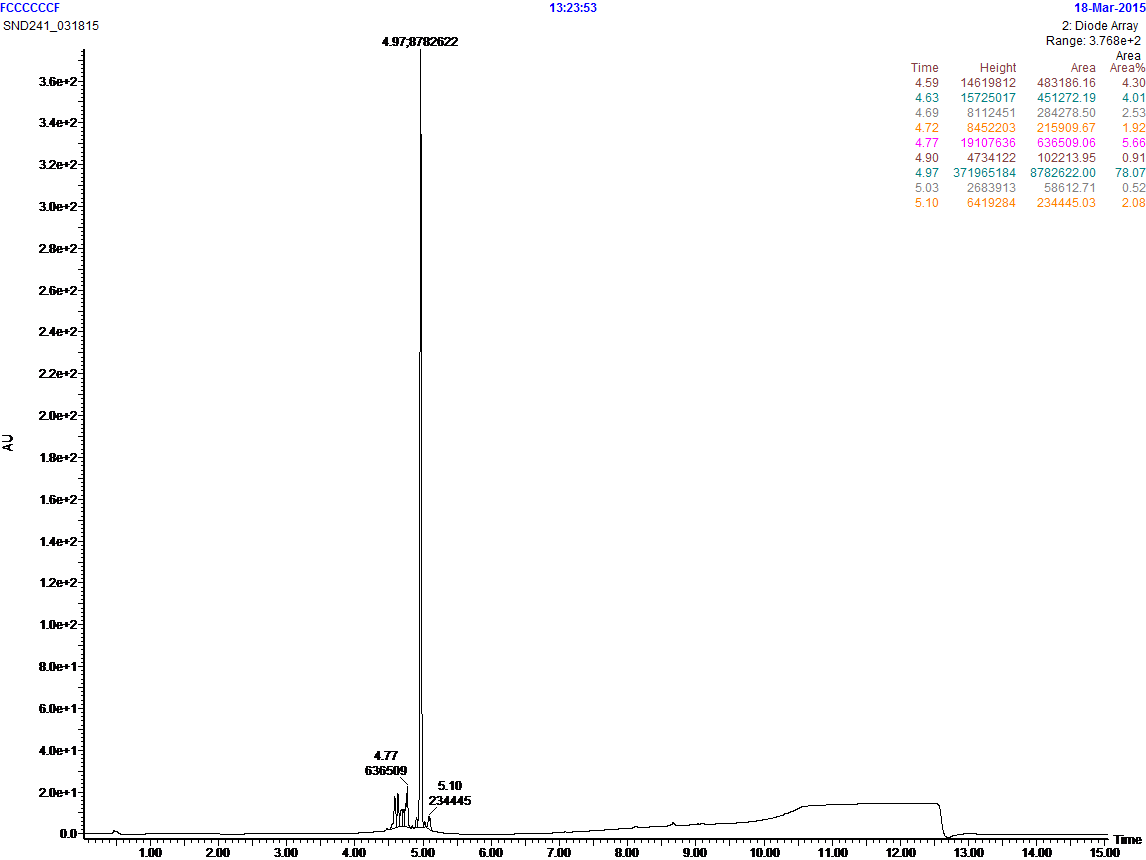


**Compound 8 -** Overall purity by LC-DAD is 78%.

**Compound 8** – Predicted MW is 1897.3. Observed MW is 1897.1.


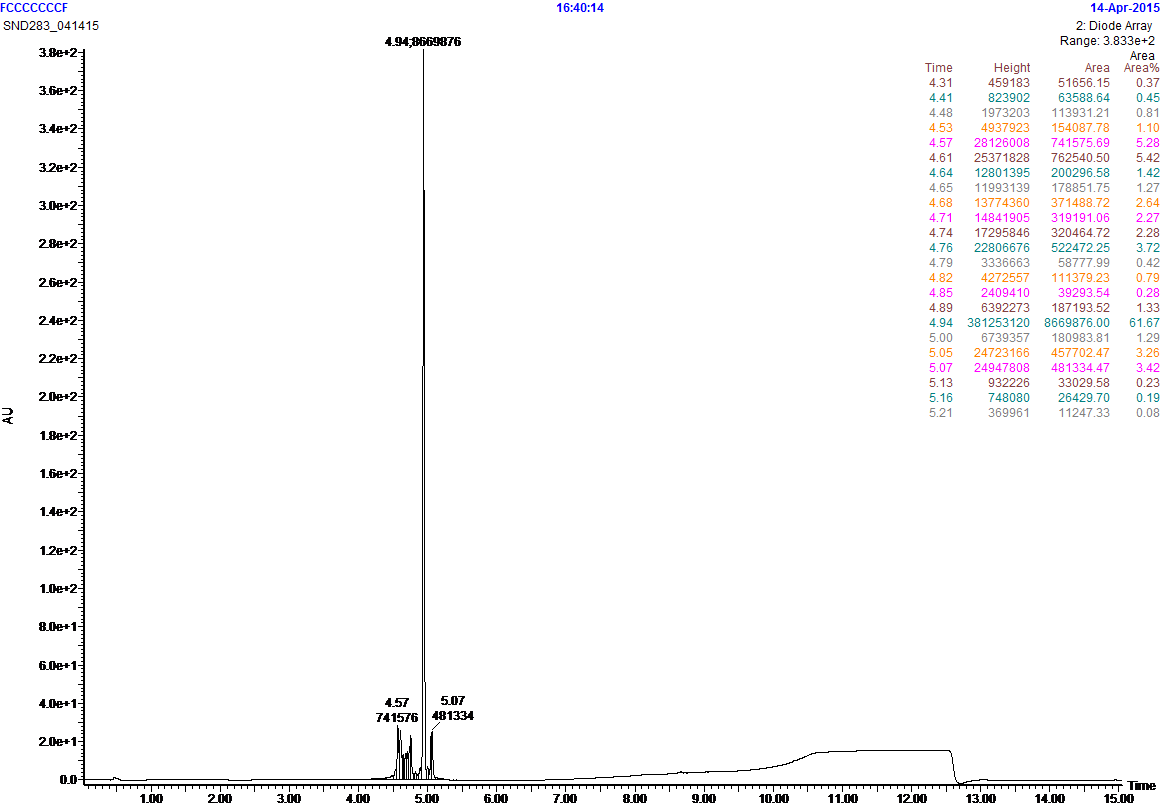


**Compound 9** - Overall purity by LC-DAD is 62%.

**Compound 9** - Predicted MW is 2021.4. Observed MW is 2021.0


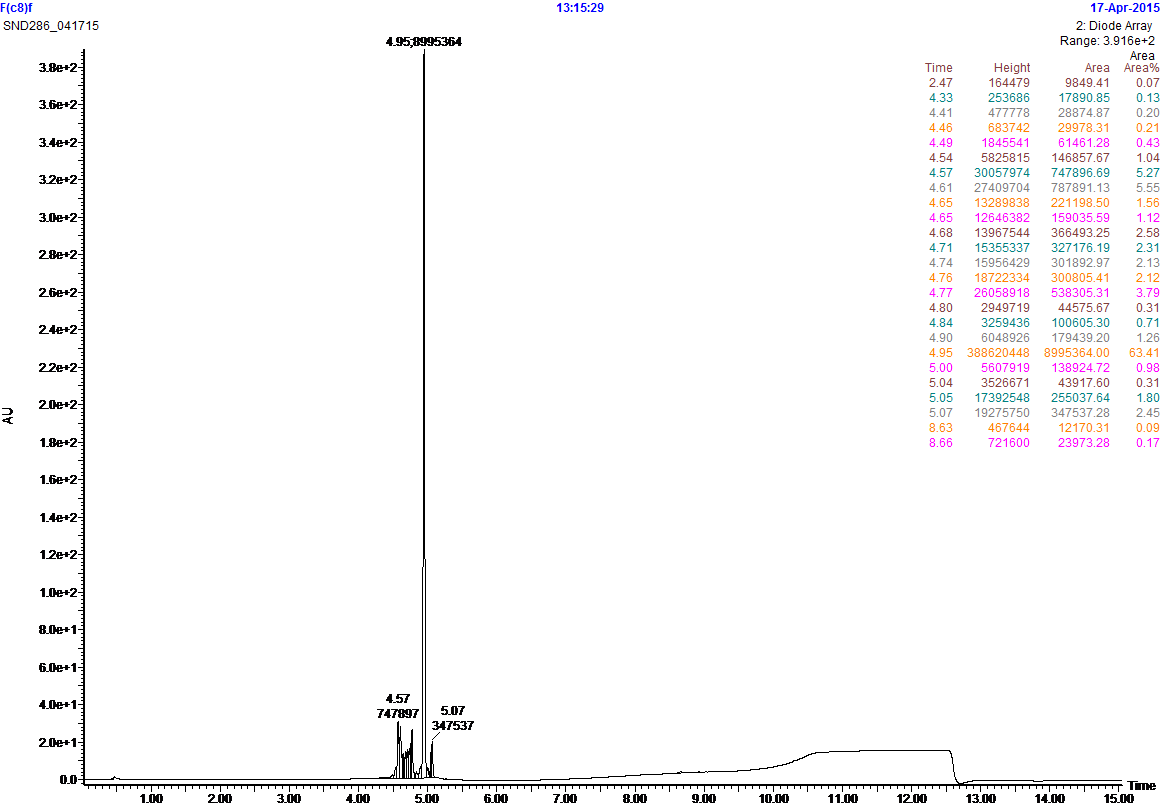


**Compound 10** – 63.4% by LC-dad.

**Compound 10** - Predicted MW is 2145.4. Observed MW is 2144.8.


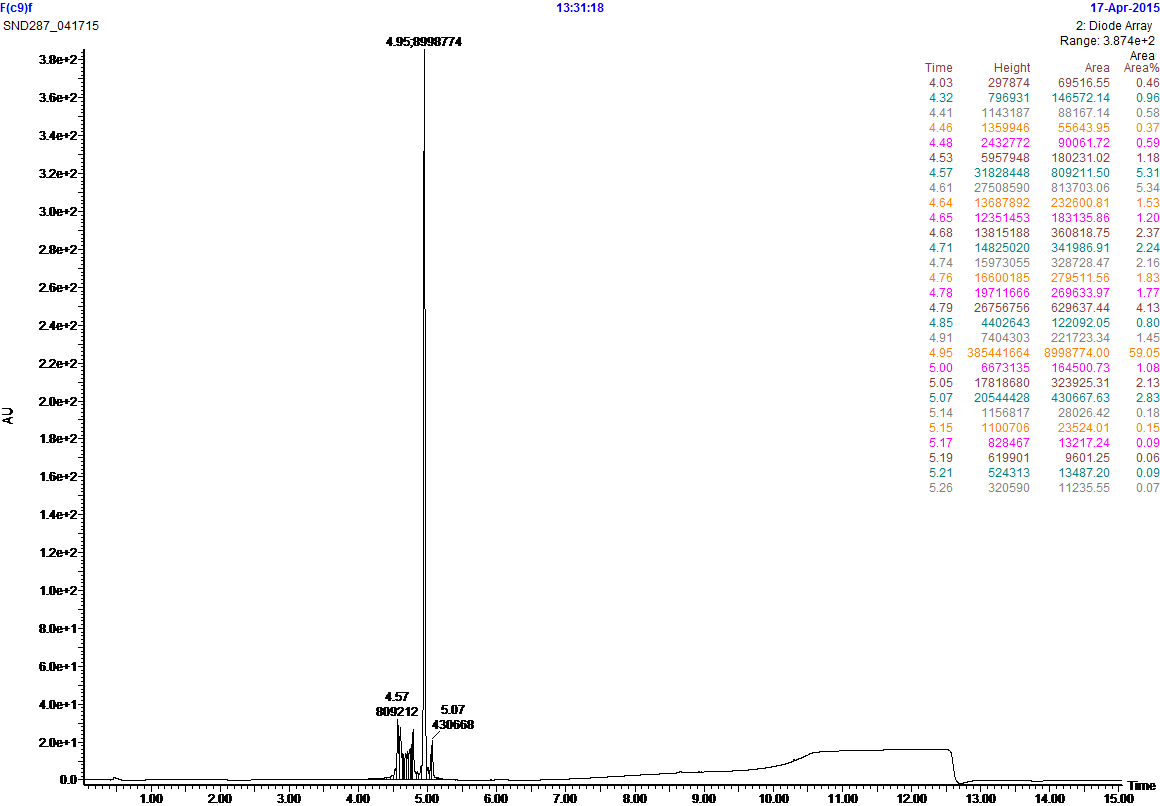


**Compound 11** - 59% by LC-DAD.

**Compound 11** - Predicted MW is 2269.5. Observed MW is 2269.0.


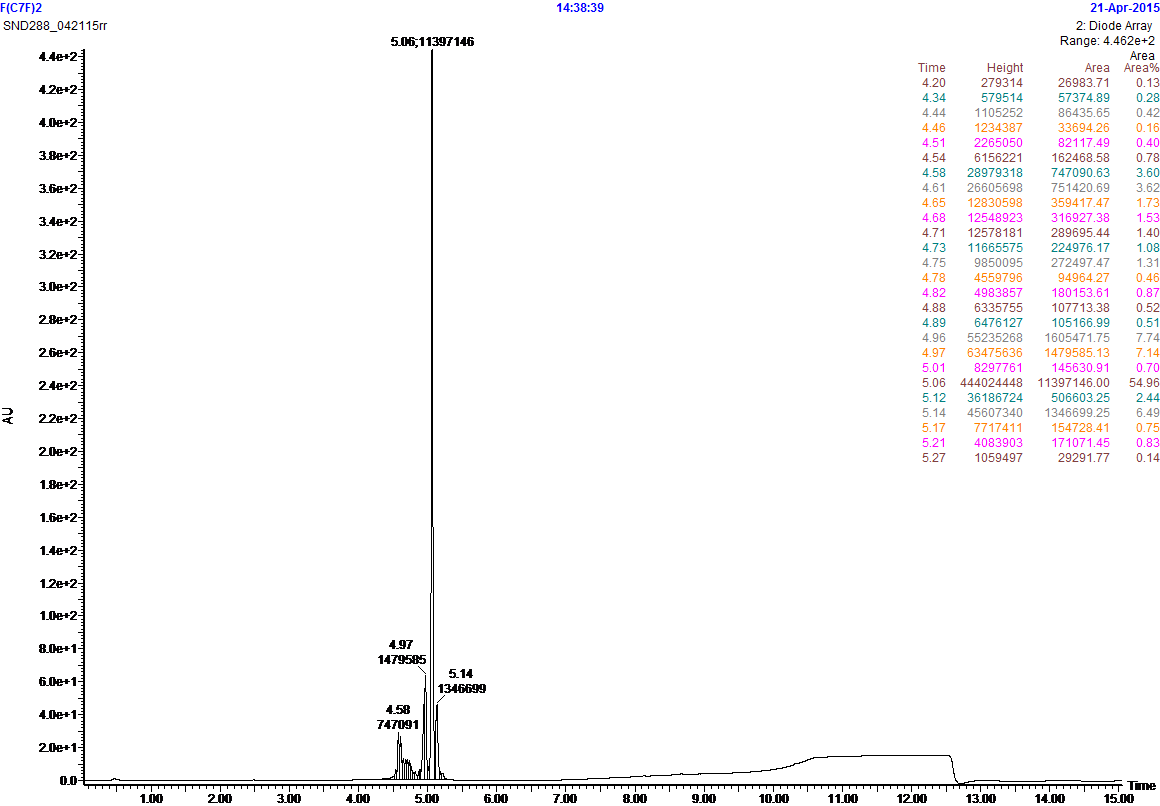


**Compound 21** - ~55% overall purity by LC-DAD.

**Compound 21** - Predicted MW is 3457.2. Observed MW is 3456.4


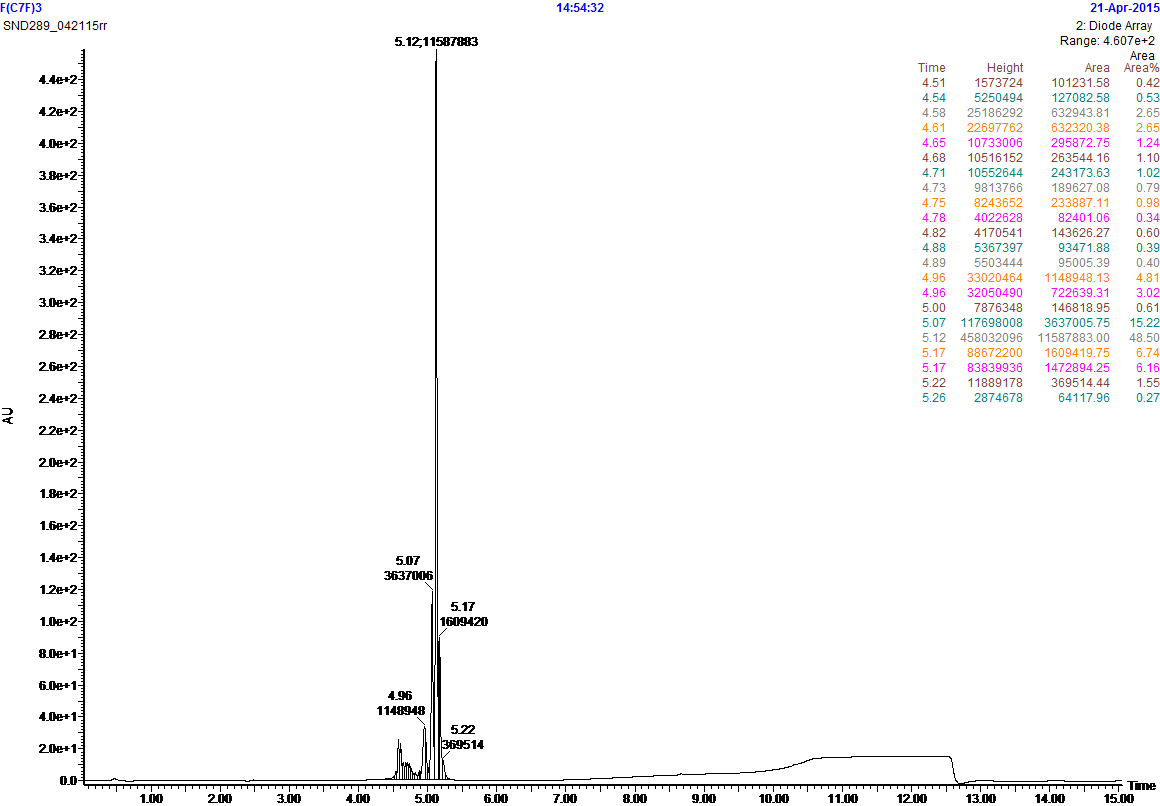


**Compound 22** - Overall purity is ~49% by LC-DAD.

**Compound 22** - Predicted MW is 4893.1. Observed MW is 4893.0.


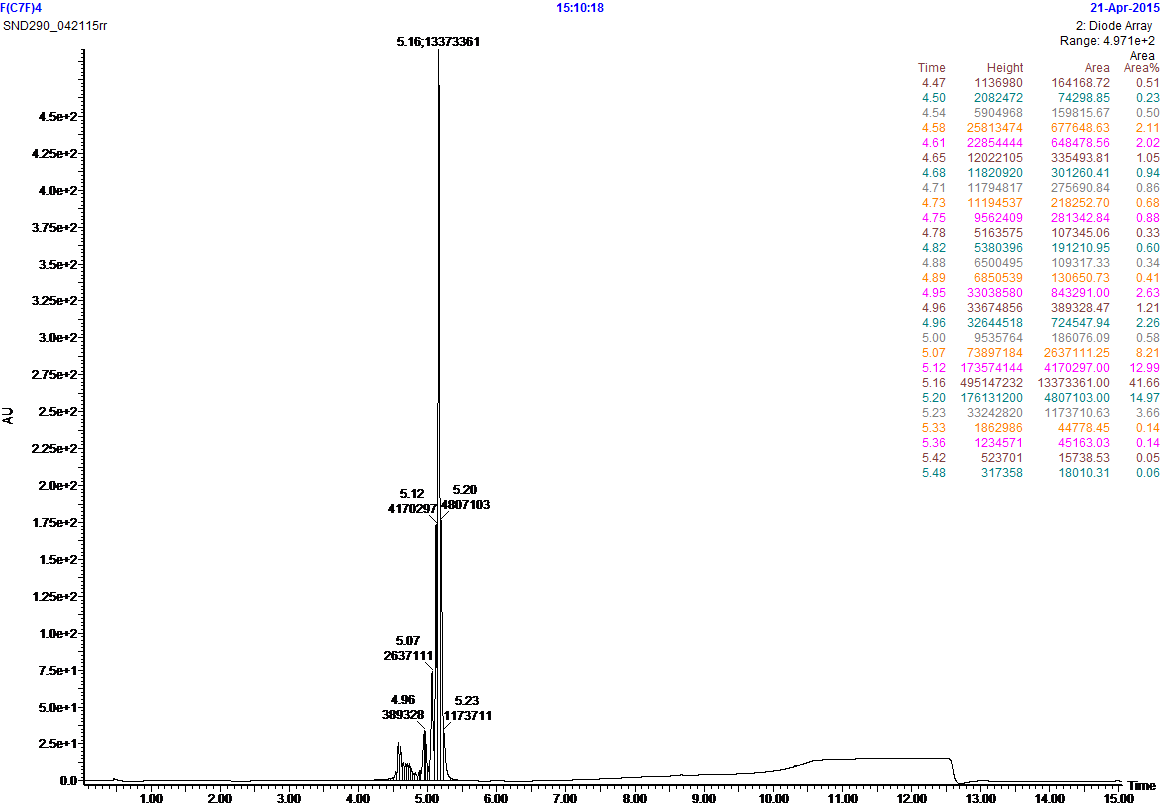


**Compound 23** - Overall purity is 42%

**Compound 23** – Predicted MW is 6328.9. Observed MW is 6329.2.


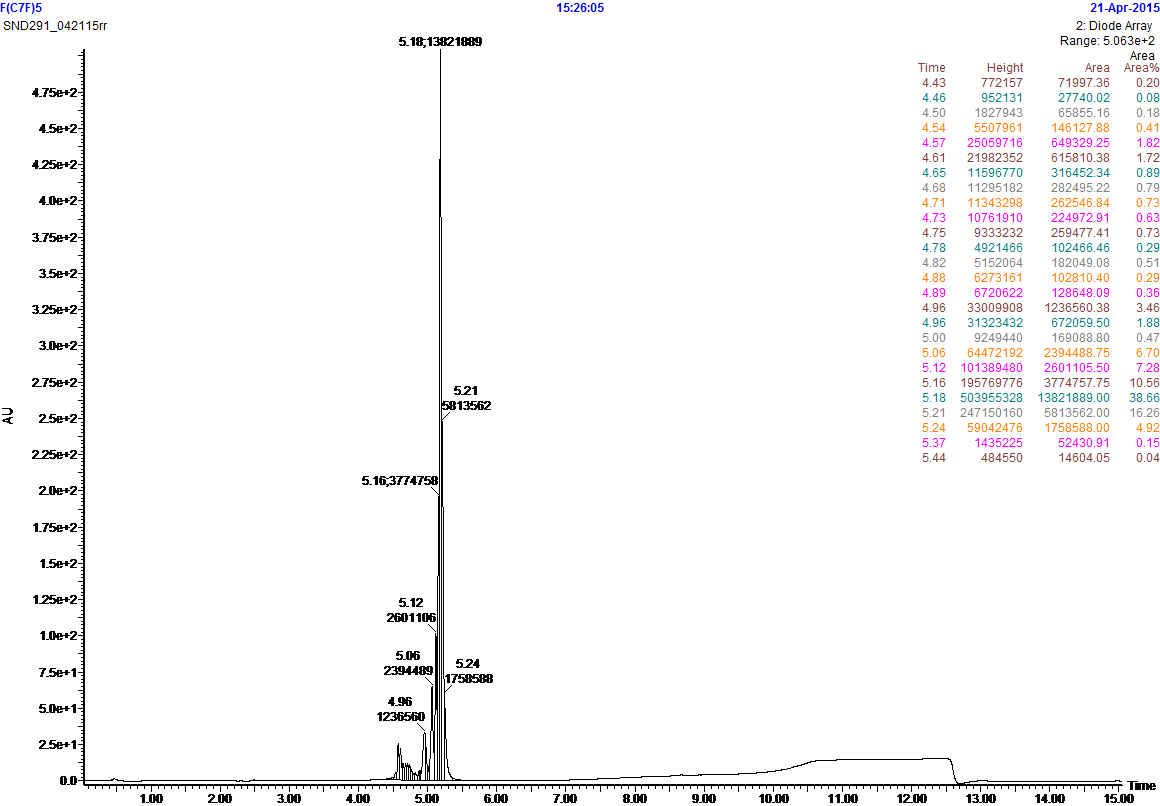


**Compound 24** - Overall purity is 39% by LC-DAD.

**Compound 24** – Predicted MW is 7764.8. Observed MW is 7765.0.


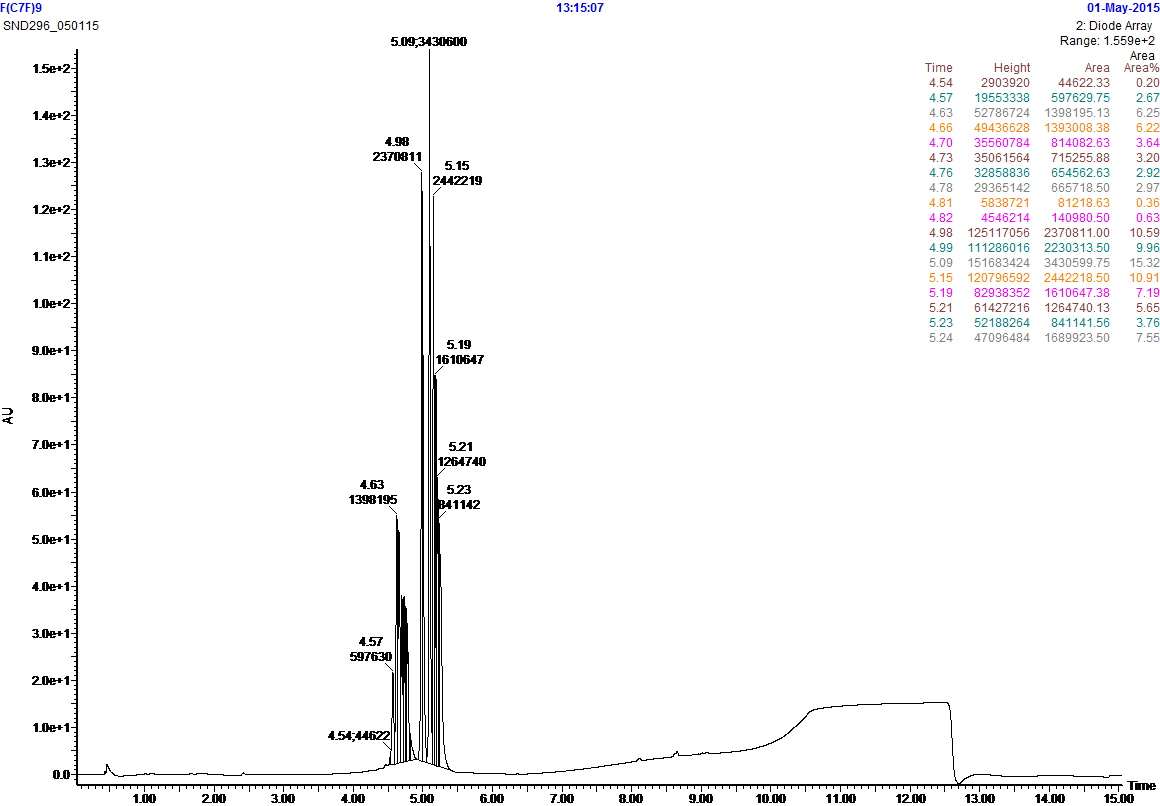


**Compound 25** - product pk is at 5.09 min. Overall purity is ~15%.

**Compound 25** - Predicted MW is 13508.2. Observed MW is 13488.6


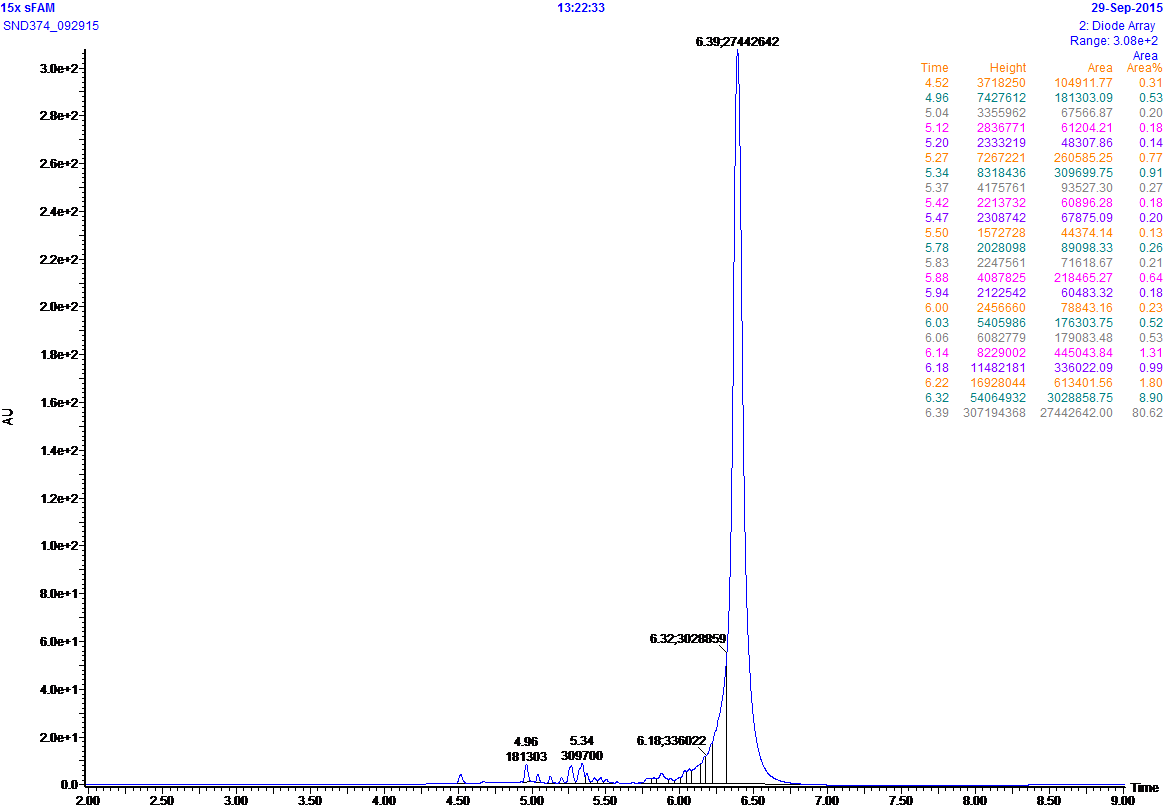


**Compound 26** – Overall purity ~80% by LC-DAD.

**Compound 26** – Predicted MW is 21404.4. Observed MW is 21402.3


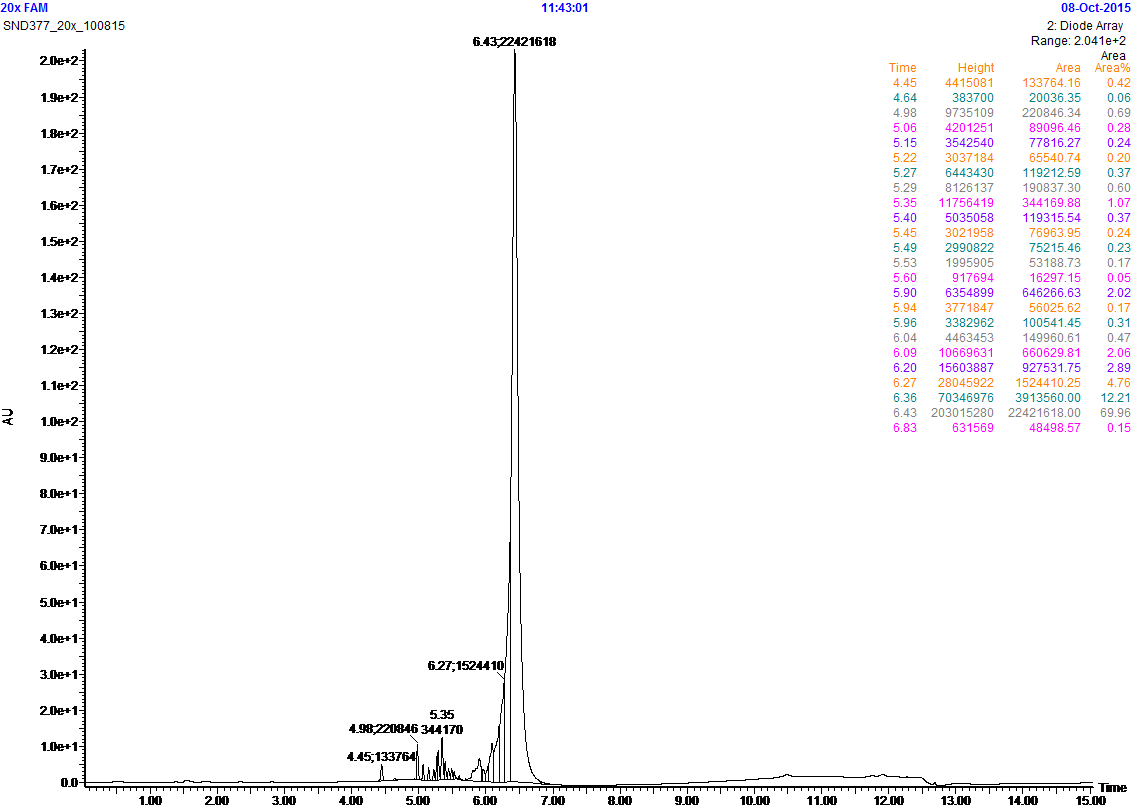


**Compound 27** – Overall purity is 70%.

**Compound 27** – Predicted MW is 28589.4. Observed MW is 28595.7

3x Pac Blue from Figure 10

main pk at 7.41m. Fully substituted. Expected MW 4844.28.

5x Pac Blue from Figure 10

main pk at 7.35m. Fully substituted BF350-SND557 would be 7923.5.

pk(s) at 7.27m. Probably dimer – would be 15581.

10x Pac Blue from Figure 10

SCR040-040-R3. Not much free dye. ~68% by LC-UV408nm, legacy HFIP.

Mostly 10x saturated, a little 9x + CNoEt.

3x AF350 from Figure 10

main pk at 7.41m. Fully substituted. Expected MW 4844.28.

5x AF350 from Figure 10

Peak at 8.03m is fully-substituted product. Expected MW 7567.

pk at 7.30m is probably dimer (would be 9424).

10x AF350 from Figure 10

MV010418-040-001-r3. SND531+BF350nhs. ~62% by Legacy HFIP method.

main pk at 7.27m. Expected MW is 15620. 15374.5 is most likely 9x+CNoEt.

3x, 5x, 10x Pac Orange from Figure 10

SCR040-024-R1-3x, -R2-5x, and -R3-10x.

040-024-r1, main pk. expected MW 5527.7.

040-024-R2, main pk. would be 9061.5.

R3, main pk. 10x would be 17896.

3x Cy3 from Figure 10

Main peak at 9.31m. Expected MW is 4941.5.

5x Cy3 from Figure 10

SCR027-006_R2 (SND498+Cy3). Fraction A3 (top) vs. A5(bottom).

De-convoluted MS of the main peak at 9.24m. Full-length, fully substituted product would be 8308.

10x Cy3 from Figure 10

SCR027-006_R3 (SND491+ Cy3). Fractions A3 v A5.

De-convoluted MS of the main peak at 8.96m. Expected MW is 18096.
