# Supplementary material for: A novel class of polymeric fluorescent dyes assembled using a DNA synthesizer
Source: PLoS One. 2020 Dec 4;15(12):e0243218. doi: 10.1371/journal.pone.0243218 (PMC7717558; doi:10.1371/journal.pone.0243218)
Supplement: Attachment — Submitted filename: Response to Reviewersv2.doc [file pone.0243218.s010.doc]

The authors would like to thank both reviewers and the editor for their time and thoughtful suggestions on making improvements to the manuscript. We have done our best to address most of the points raised in the revised manuscript. Specifically addressing each point raised:

Editor:

a) All figures (and supplementary information) redone to increase resolution and clarity except for the 3 structural in silico simulation figures (they would have to be completely re-simulated and computing time in Japan would be difficult to obtain given the time frame). Also, we believe the images convey the overall macromolecular structure well in our opinion).
b) Added new **Supplementary Information (SM1,SM2,SM7,SM8 and SM9)** = mass spec data for oligomers and polymers in Figures (2,5 and 10) also included are purity levels of most of the constructs (**SM9**). Changed some “polymer” references to “oligomer” for the shorter constructs. Added statement on scalability of these types of compounds (line 217-218).

Journal Requirements:

1. All updated figures and SMs were passed through PACE
2. Data availability statement amended as requested. Raw data in the form of excel spread sheets is provided for all emission curves in Figures 5,6 and 10 (SM8). LCMS data for the constructs is included as well.
3. Modified the Funding statement to include:

“The funder provided support in the form of salaries for all authors, but did not have any additional role in the study design, data collection and analysis, decision to publish, or preparation of the manuscript. The specific roles of these authors are articulated in the ‘author contributions’ section.”

Also modified author contributions as requested.

4. Modified the Competing Interests Statement to include:

"The commercial affiliation does not alter our adherence to PLOS ONE policies on sharing data and materials.”

Reviewer 1:

1. All figures (and supplementary materials) redone to increase resolution and clarity except for the 3 structural in silico simulation figures (they would have to be completely re-simulated and computing time in Japan would be difficult to obtain given the time frame). Also, we believe the images convey the overall macromolecular structure well in our opinion). Per reviewers and editor requests
2. Added new **Supplementary Information (SM1,SM2,SM7 and SM9)** = mass spec data and HPLC chromatograms for oligomers and polymers in Figures (2,5 and 10) (yield data not possible for microscale synthesis but is provided for larger scale synthesis along with purity data in **SM5**. Purity data also included in **SM9**.
3. Modified title and changed some “polymer” references to “oligomer” for the shorter constructs. Many of the constructs are over 50 residues long which we left as polymer
4. Added additional x axis and y axis legends to Figures.
5. The suggestion made to try and qualitatively analyze the quenching efficiency based on distance calculations while valid, would be difficult due to the computation time required as mentioned above. We feel the images and emission data in the figures (2 and 6) are sufficient to give readers an understanding of the linear control of quenching in this system.
6. Added mention of protein based fluorescent compounds and potential role of sodium ions in Figure 8.
7. Minor edits as pointed out by reviewers for added clarity

Reviewer 2:

a. All figures (and supplementary materials) redone to increase resolution and clarity except for the 3 structural in silico simulation figures (they would have to be completely re-simulated and computing time in Japan would be difficult to obtain given the time frame). Also, we believe the images convey the overall macromolecular structure well in our opinion). Per reviewers and editor requests
b. Added statement on scalability of these types of compounds (line 217-218)
c. Added an additional **Figure (1)** – coupling chemistry scheme -
d. Added brief text description of chemical steps – lines 58-66
e. Added new reference (7) Caruthers paper for original published report of phosphoramidite chemistry
